# Secreted Frizzled-Related Protein 5 Protects Against Cardiac Rupture and Improves Cardiac Function Through Inhibiting Mitochondrial Dysfunction

**DOI:** 10.3389/fcvm.2021.682409

**Published:** 2021-09-09

**Authors:** Xin Huang, Yan Yan, Wen Zheng, Youcai Ma, Xiao Wang, Wei Gong, Shaoping Nie

**Affiliations:** ^1^Emergency & Critical Care Center, Beijing Anzhen Hospital, Capital Medical University, Beijing, China; ^2^Beijing Institute of Heart, Lung, and Blood Vessel Diseases, Beijing, China

**Keywords:** secreted frizzled-related protein 5, myocardial infarction, mitochondrial dysfunction, coronary heart disease, AMPK

## Abstract

**Background:** Secreted frizzled-related protein 5 (Sfrp5) has been suggested to be a protective regulatory protein in coronary heart disease. However, the role of Sfrp5 in regulating ischemic injury and its consequences is not known. The aim of our study was to explore the effects of Sfrp5 on hearts after myocardial infarction (MI) and to investigate the underlying mechanisms.

**Methods and Results:** We found that Sfrp5 was downregulated over time in the heart tissue of MI mice. To further elucidate the role of Sfrp5 during MI, we established a cardiac overexpression of an Sfrp5 mouse model using the cardiotropic adeno-associated virus serotype 9 (AAV9). Overexpression of Sfrp5 significantly reduced infarct size as demonstrated by a decrease in mortality owing to cardiac rupture. Moreover, cardiac overexpression of Sfrp5 increased left ventricular function and mitochondrial biogenesis, decreased cardiomyocyte apoptosis, suppressed inflammation reaction, inhibited oxidative stress, and ameliorated cardiac remodeling as demonstrated by left ventricular ejection fraction, mitochondrial morphology, heart weight, NADH oxidase activity levels, and myocardial fibrosis at 2 weeks post-MI. At the molecular level, overexpression of Sfrp5 significantly increased the expression of p-AMPK^Thr172^ protein with higher expression of mitochondrial fusion protein (MFN1 and MFN2) and lower expression of mitochondrial fission protein (p-Drp1^Ser616^/Mid49/MFF/Fis-1). In isolated neonatal rat cardiac myocytes, Sfrp5 treatment attenuated hypoxia-induced mitochondrial dysfunction. Inhibition of AMPK activity with compound C abrogated this benefit.

**Conclusions:** Sfrp5 overexpression inhibits ischemic injury, reduces risk of cardiac rupture, ameliorates post-MI remodeling, and decreases the progression to heart failure via disrupting mitochondrial dysfunction and partly through normalizing the AMPK activity.

## Introduction

Cardiovascular disease is the leading cause of death and disability in the world (1). Myocardial infarction (MI) is a major event of cardiovascular disease and associated with high morbidity. In heart tissue, MI causes excessive loss of cardiac myocytes, and a progressive cardiac remodeling ultimately results in cardiac dysfunction and heart failure (HF) (2). Despite significant advances in reperfusion strategy and pharmacological treatment of MI in recent decades, the mortality rate caused by MI remains extremely high.

Although the pathogenesis of MI is multifactorial, mitochondrial dysfunction is thought to be one of the major causes (3). Mitochondria as an organelle for translating energy can regulate metabolism and ATP production and thus sustain cardiomyocyte contractility and function in normal myocardial tissue (4). However, in pathological condition, impaired mitochondria alter cardiac energy metabolism, decrease cardiac efficiency, and worsen the progression of cardiac remodeling (5). Damaged mitochondria release reactive oxygen species (ROS) into the cytoplasm, resulting in cell apoptosis and aggravated cardiac remodeling post MI (6). Recently, Wnt signaling has been acknowledged to play an important role in mitochondrial energetic metabolism (7).

Secreted frizzled-related protein 5 (Sfrp5), a member of the Sfrps family, restricts Wnt signaling through inactivate specific Wnt5a actions and participates in mitochondrial metabolic function (8). Sfrp5 inactivated the activity of NADPH oxidases and decreased vascular oxidative stress (9). Inhibition of NADPH oxidase decreased the generation of mitochondrial ROS, improved mitochondrial dysfunction, and further ameliorated maladaptive cardiac remodeling in HF (10, 11). Recently, significant concerns have been raised over the effects of Sfrp5 on heart development and oxidative metabolism, which were associated with the pathogenesis of ischemic cardiovascular disease (8, 9, 12, 13). Nakamura et al. demonstrated that Sfrp5 significantly reduced inflammatory responses after ischemia/reperfusion and improved cardiac function (14). Given that Sfrp5 ameliorated the development for a host of disease states that are associated with significant cardiovascular disease (13, 15). We felt it was critical from a mechanical perspective to clarify the role of Sfrp5 in the ischemic heart. The aim of our study is to explore the role of Sfrp5 in mitochondrial dysfunction and cardiac injury after MI.

## Materials and Methods

### Adeno-Associated Virus Serotype 9 (AAV9) Vector Delivery

AAV9 expressing a full-length Sfrp5 cDNA (AAV9/Sfrp5) and a scramble control (AAV9/NC) was generated by Vigene Biosciences Co., Ltd. (Shandong, China), according to the manufacturer's protocol. The cDNA was constructed by encoding N-terminally Flag-tagged Sfrp5 with the CMV promoter. The sequence of AAV9/Sfrp5 is available in the online-only data supplement. C57BL/6J male mice (6–8 weeks old) were purchased from Hua Fu Kang Biotechnology Co., Ltd. (Beijing, China). Animals had free access to food and water and were kept in polycarbonate cages under specific pathogen-free conditions with a 12-h light/dark cycle. The animal study was reviewed and approved by the Animal Subjects Committee of Capital Medical University, in accordance to the Guide for the Standard Animal Welfare Regulations of Capital Medical University, and all procedures conformed to the guidelines from Directive 2010/63/EU of the European Parliament on the protection of animals used for scientific purposes or the NIH Guide for the Care and Use of Laboratory Animals.

A total of 180 animals were randomly divided into two groups of 90 animals each, as follows: (i) C57BL/6 WT mice with AAV9-NC injection and (ii) C57BL/6J wild-type (WT) mice with AAV9-Sfrp5 injection. The adenovirus was diluted into sterile water with 5 ×10^10^ viral genomes (vg)/μl, and 10 μl was injected into the tail vein of mice using 1-ml syringes. The presence of the Sfrp5 was confirmed by qPCR and western blot before the experiments.

### Cardiac MI Model

Four weeks after injection, AAV9-NC and AAV9-Sfrp5 C57BL/6J male mice were used to induce MI according to the established mouse model of MI (16). Briefly, mice received continuous gas anesthesia with 2% isoflurane (YiPin Pharmaceutical, China), and the left side of their chest was opened. A 7.0 nylon suture ligature was tied at the left anterior descending coronary artery to totally occlude the blood flow. The establishment of the MI model was suggested to be successful when the anterior wall of the left ventricle (LV) appeared pale. The sham operation was performed with a similar procedure only without ligating the coronary artery. The heart was instantly put back into the thorax. The muscle and skin were sutured. All surgeries and subsequent analyses were conducted in a blinded manner. A total of 180 mice were used in the present study, and 38 died within 12 h due to operation reasons. We divided the surviving mice into four groups as follows: (i) AAV9-NC mice with sham operation; (ii) AAV9-Sfrp5 mice with sham operation; (iii) AAV9-NC mice with MI operation; and (iv) AAV9-Sfrp5 mice with MI operation. In each group, 20 animals were used for survival rate study. The rest of animals were used for TTC staining, mitochondrial function tests, histology, RT-PCR, and western blot analysis. Two weeks after MI, mice were analyzed for morphometrics. Lung weight (LW), heart weight (HW), body weight (BW), and tibia length (TL) were determined, and LW/BW, HW/BW, and HW/TL (the primary morphometric measures of HF and hypertrophy) were calculated.

### TTC Staining

TTC staining was conducted to assess the infarct size in heart tissue, as previously described (17). Briefly, at 24-h post coronary artery ligation, mice were anesthetized with tribromoethanol (20 mg/0.2 ml, T48402, Sigma-Aldrich), and heart tissues were rapidly removed from mice. After being washed with ice-cold saline, the heart tissues were frozen at −20°C for 30 min and cut into four transverse slices of equal thickness (approximately 2 mm) from the apex to base. The slices were then immersed in 2% TTC (G3005, SolarBio) at 37°C in the dark for 15–30 min. After staining with TTC, the slices were photographed and used to assess the infarct size by ImageJ software (National Institutes of Health, Bethesda, MD, United States). The percentage of heart infarct size (%) = infarct volume (unstained areas)/total volume of transverse slices × 100%.

### Echocardiography

Tribromoethanol is commonly used in the field of cardiovascular research due to its modest cardiodepressive effects, relatively short duration of action, and less non-controlled status. Compared to isoflurane and pentobarbital sodium, tribromoethanol has the least effect on heart function (18). Therefore, mice were anesthetized with an intraperitoneal injection of tribromoethanol (20 mg/0.2 ml, T48402, Sigma-Aldrich). The *in vivo* cardiac function of mice was measured via transthoracic echocardiography using a Vevo 2100 high resolution imaging system with a 30-MHz transducer (GE Healthcare, United States). M-mode images were captured to measure the left posterior ventricular wall and interventricular septum thickness. Complete systolic and diastolic indexes were measured at 0, 7, and 14 days following MI.

### Histology

Paraffin-embedded ventricular tissue was cut into 5-μm-thick sections and mounted onto poly-l-lysine treatment slides. All procedures were performed at room temperature. Hematoxylin-and-eosin (HE) staining of rehydrated ventricular sections was performed using an HE staining kit (G1120, SolarBio). For areal measurement, the cardiomyocyte was circled by the lasso tool, and then the areas of the circles were calculated and converted to their actual values using the scale bar by the NIS-ELEMENTS quantitative automatic program (Nikon) in the HE-stained heart sections. A total of 200 cardiomyocytes were taken for relevant statistics. Fibrosis in the ventricular sections was stained using Masson's Trichrome Stain Kit (G1340, SolarBio). Fibrosis from stained ventricular sections was imaged on an Axio Imager M1 polarized light microscope, and the areas of myocardium fibrosis was determined by binarizing images before analyzing. The fibrosis areal fraction of the myocardium was determined by dividing the area of fibrosis by the total tissue area of each image.

### Transmission Electron Microscopy (TEM)

Fresh isolated LV tissue was fixed with 2.5% glutaraldehyde in 0.1 M phosphate buffer overnight at 4°C. After fixation with 1% osmium tetroxide in 0.1 M phosphate buffer, the tissue was dehydrated in an ethanol series and infiltrated with propylene oxide to embed in Epon 812 resin. Ultrathin sections were cut with an ultramicrotome, post-stained with uranyl acetate and lead citrate, and then observed by TEM (Quemesa; Olympus, Tokyo, Japan).

### Cell Culture and Isolation of Cardiac Cells

Neonatal rat cardiac myocytes (NRCMs) were isolated from 1–3-day-old male Sprague-Dawley (SD) rats. All operations were performed in a sterile environment. After the hearts were removed, they were minced into approximately 1-mm^3^ pieces with scissors and dissociated into single cells via incubation in a 0.25% trypsin solution (C0201, Beyotime) in 25–30 times for 3–5 min each time. The liquid from the first digestion was discarded, and the remaining dispersed cells were incubated on 15-cm culture plates for 120 min at 37°C with 5% CO_2_. The cardiomyocytes were collected, resuspended, and transferred to six-well plates and cultured in DMEM/F12 with 10% fetal bovine serum (FBS), 100 U/ml penicillin, and 100 μg/ml streptomycin for 4 days prior to experiments. To establish the hypoxia model, we placed the cells in an anaerobic condition with an anoxia bag for 12 h in the presence or absence of 1 μM recombinant Sfrp5 protein (7195-SF-050, R&D).

### Determination of ATP Content and NAD^+^/NADH Ratio

Myocardial tissue ATP content was determined by an ATP Assay Kit (Abcam, ab 83355) according to the manufacturer's instructions. Concisely, the tissues were homogenized in an ATP Assay Buffer with a Dounce homogenizer sitting on ice, with 20–25 passes, and the sample was centrifuged for 2–5 min at 4°C with 13,000 *g* using a cold microcentrifuge to remove any insoluble material. To remove the enzymes that can interfere with the assay from the sample, we used a Deproteinizing Sample Preparation Kit-TCA (Abcam, ab204708). Then, a reaction mix was added into the prepared sample, and the homogenates were incubated for 30 min. ATP was quantified by fluorometric (Ex/Em = 535/587 nm) methods and calculated by ATP standard curve. NAD^+^/NADH was measured using heart cytosolic fractions by a NAD^+^/NADH Assay Kit (Abcam, ab65348) as described previously (19). Briefly, the tissues were homogenized in a NADH/NAD^+^ Extraction Buffer for extraction of total NAD^+^ and NADH. A single aliquot of extracted samples was heated at 60°C for 30 min. Under these conditions, all NAD^+^ will be decomposed, while NADH will still be intact. Next, the reaction mix was added to each sample well, and the plate was incubated at room temperature for 5 min to convert NAD^+^ to NADH. Finally, NADH Developer was added into each well. A reading at 450 nm of a working reagent mix containing total NAD^+^/NADH and NADH alone was taken after a reaction cycle at room temperature for 1 h. Calculation of total NAD^+^/NADH and NADH alone was performed using a NADH standard curve.

### Western Blot

Heart tissues after 14 days of MI were washed with cold PBS, lysed with the mixture of RIPA buffer (SolarBio, China, Cat. No: R0020) and Protease Inhibitor Cocktail (Thermo Fisher Scientific, Waltham, MA, 87786). Lysates were centrifuged at 12,000 *g* for 20 min at 4°C. The protein concentration of ventricular extracts was measured using a Pierce BCA Protein Assay Kit (Thermo Fisher Scientific, Waltham, MA, 23225). The total extract protein from mouse ventricular extracts (60 μg) was separated on 10% and 12% polyacrylamide gels and then wet-transferred onto polyvinylidene difluoride membranes (Millipore). Membranes were immunoblotted with primary monoclonal antibodies against AMPK (1:1,000 dilution, 2532S, CST), p-AMPK^Thr172^ (1:1,000 dilution, 2535S, CST), Bcl-2 (1:1,000 dilution, 26593-1-AP, ProteinTech), Bax (1:1,000 dilution, 50599-2-Ig, ProteinTech), a-tubulin (1:1,000 dilution, 11224-1-AP, ProteinTech), MFN1 (1:1,000 dilution, 13798-1-AP, ProteinTech), MFN2 (1:1,000 dilution, 9482S, CST), Fis-1 (1:1,000 dilution, ab71498, Abcam), p-Drp1^Ser616^ (1:1,000 dilution, AF8470, Affinity), Drp1 (1:1,000 dilution, DF7037, Affinity), SIRT1 (1:1,000 dilution, DF6033, Affinity), MiD49 (1:1,000 dilution, DF12044, Affinity), MFF (1:1,000 dilution, 14090-1-AP, ProteinTech), and Sfrp5 (1:1,000 dilution, PA5-71770, Invitrogen). α-Tubulin was used as loading controls for mouse ventricular extracts. Protein bands were detected using IRDye® 680RD goat anti-rabbit IgG Secondary Antibody (1:5,000 dilution, LI-COR, 926-68071).

### Measurement of Mitochondrial Oxygen Consumption Rate (OCR)

The OCR was monitored in real time with a Seahorse XF-24 extracellular flux analyzer (Seahorse Bioscience, Houston, TX, USA). One day before the experiment, the sensor cartridge was placed into the calibration buffer (Seahorse Bioscience) and incubated at 37°C in a non-CO_2_ incubator overnight. The assay medium (pH 7.4) was prepared with a glucose solution (1 M), pyruvate solution (100 mM), and glutamine solution (200 mM) before measurement. NRCM cells were plated at 5 × 10^5^ cells/well in a Seahorse 24-well microplate (Seahorse Bioscience) and cultured in anaerobic or normal conditions for 12 h in the presence or absence of 1 μM recombinant Sfrp5 protein, respectively. Next, the medium was removed, and NRCM cells were incubated in an XF assay medium. The mitochondrial complex inhibitors (oligomycin, FCCP, and rotenone/antimycin-A) provided by XF Cell Mito Stress Test kit (Seahorse Bioscience) were freshly prepared in XF assay media. During the experiment, oligomycin (2.5 μM) was injected into each well at 50 min, followed by FCCP (1 μM) at 74 min, rotenone/antimycin A (2.5 μM) at 98 min. Oligomycin acts as an ATP synthase inhibitor, and therefore OCR reduction after oligomycin treatment represents ATP turnover. FCCP is a mitochondrial uncoupler and dissipates the electrochemical proton gradient to induce the maximum respiration termed as respiratory capacity or uncoupled respiration. Rotenone/antimycin-A as a complex I/III inhibitor paralyzes ETC, and mitochondrial respiration goes down to zero.

The Kaplan–Meier survival curves with the log-rank test was performed to compare the 14-day survival rate of all animals. The mortality data were analyzed by the chi-square tests. Quantitative data are expressed as mean ± SD and obtained from at least three independent experiments. Data were tested for normal distribution using the Shapiro–Wilk test by statistical software SPSS 25.0 (IBM Corp., Armonk, NY). Student's *t*-test or one-way ANOVA was performed for normally distributed data. Otherwise, non-parametric tests were used prior to one-way ANOVA (GraphPad Prism 7 software, La Jolla, CA). A *p*-value < 0.05 was considered statistically significant. An expanded Methods section is available in the online-only data supplement.

## Results

### Overexpression of Sfrp5 Decreases Post-MI Mortality and Cardiac Rupture

**Figure 1:**
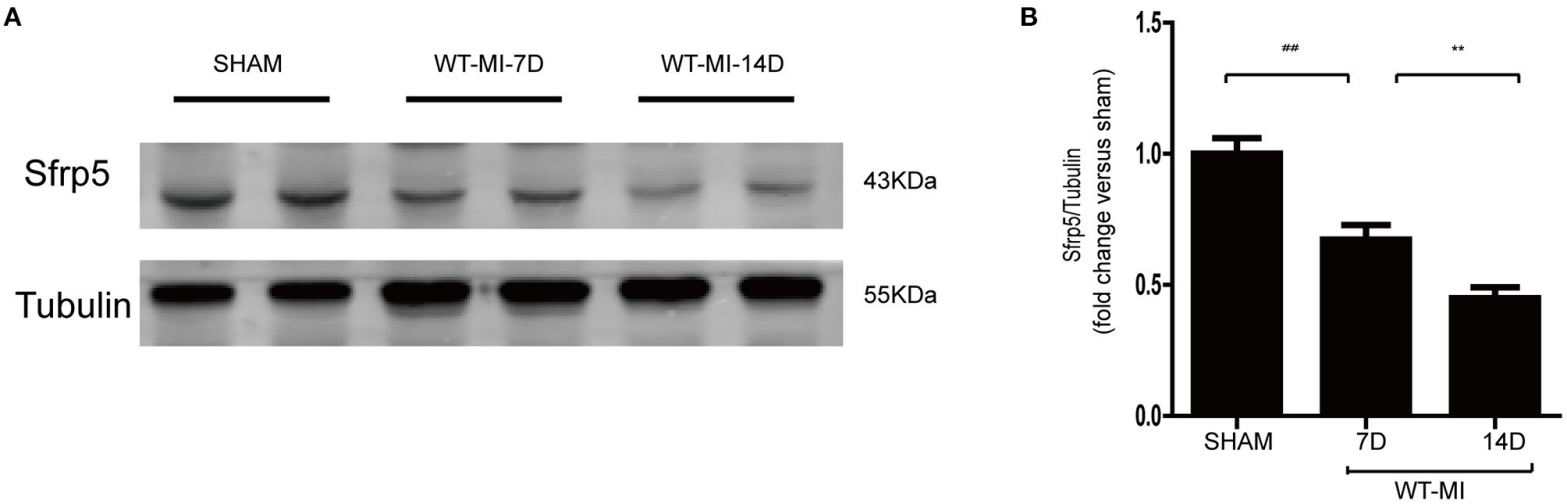
Expression levels of Sfrp5 in hearts after MI. **(A)** Protein expression of Sfrp5 in mouse hearts at 7 and 14 days after MI. **(B)** Quantitative analysis of the relative Sfrp5 protein expression. Tubulin was used as a loading control (values are presented as mean ± SD, *n* = 6 per group). ^##^*p* < 0.01 mice at 7 days after MI vs. mice at 7 days after sham operation. **p < 0.01 mice at 14 days after MI vs. mice at 7 days after MI.

Temporal changes in regional Sfrp5 expression have not previously been investigated in mice after MI. Therefore, we quantified the expression of Sfrp5 taken from C57BL/6J mice after MI at the border zone in the heart at 7 and 14 days. The expression of Sfrp5 was significantly decreased over time in heart tissues of MI as detected by western blot (Figure 1). MI caused a decrease in Sfrp5 expression in a time-dependent manner.

**Figure 2:**
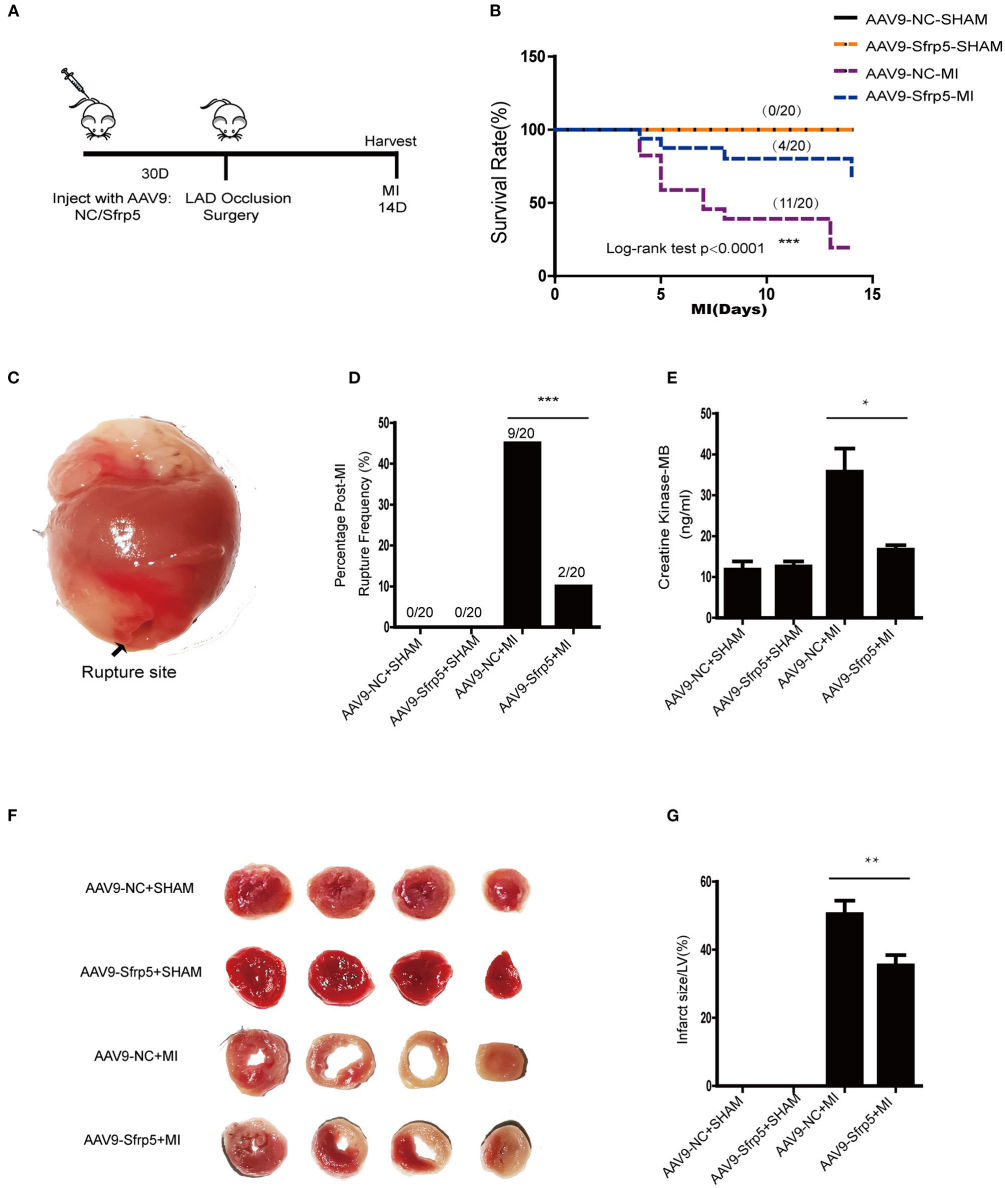
Effects of Sfrp5 on survival and cardiac rupture rate in mice at 2 weeks after MI. **(A)** Overview of the method for AAV9 injection and the mouse models for MI. **(B)** Survival rate at 14 days after MI (*n* = 20 per group). **(C)** Representative image of rupture site. **(D)** Cardiac rupture rate at 2 weeks post infarction (*n* = 20 per group). **(E)** The level of CK-MB at 24 h post infarction (values are presented as mean ± SD, *n* = 6 per group). **(F)** Representative images of heart sections isolated at 24 h post infarction and stained with TTC. **(G)** Quantitative analysis of the percentage of infarct size (values are presented as mean ± SD, *n* = 6 per group). *p < 0.05 AAV9-NC-MI mice 1 day after MI vs. AAV9-Sfrp5-MI mice at 1 day after MI. **p < 0.01 AAV9-NC-MI mice 1 day after MI vs. AAV9-Sfrp5-MI mice at 1 day after MI. ***p < 0.001 AAV9-NC-MI mice 14 day after MI vs. AAV9-Sfrp5-MI mice at 14 day after MI.

To elucidate whether Sfrp5 was detrimental or beneficial for post-MI cardiac injury, Sfrp5 overexpression was induced in mice by intravenous injection of AAV9-Sfrp5 or AAV9-NC through the tail vein 4 weeks before MI or sham operation (Figure 2A). Compared with AAV9-NC, AAV9-Sfrp5 mice had significantly decreased mortality post MI (20 vs. 55% for littermate controls; *p* < 0.0001) (Figure 2B). Postmortem examination revealed that cardiac rupture was the main cause of deaths (10 vs. 45% for littermate controls; *p* < 0.0001) (Figure 2C). Given the early decrease in rupture (Figure 2D), we next asked whether ischemic injury was also decreased in the AAV9-Sfrp5 mice. To assess this, we examined the levels of CK-MB at 24 h post coronary artery ligation from the plasma of mice that were sacrificed for TTC staining. We found that the level of plasma CK-MB was indeed significantly decreased in AAV9-Sfrp5 than in AAV9-NC mice (Figure 2E). Furthermore, we examined the infarct size at 24 h post coronary artery ligation. The infarct size was also significantly decreased in AAV9-Sfrp5 than in AAV9-NC mice (Figures 2F,G). The presence of the Sfrp5 was confirmed by qPCR and western blot before the experiments. The expression level of Sfrp5 was significantly higher in AAV9-Sfrp5 mice than in AAV9-NC mice (online-only data Supplementary Figures 1A–C).

### AAV9-Sfrp5 Ameliorates LV Functional Deterioration, Pathological Hypertrophy, and Fibrosis Following MI

**Figure 3:**
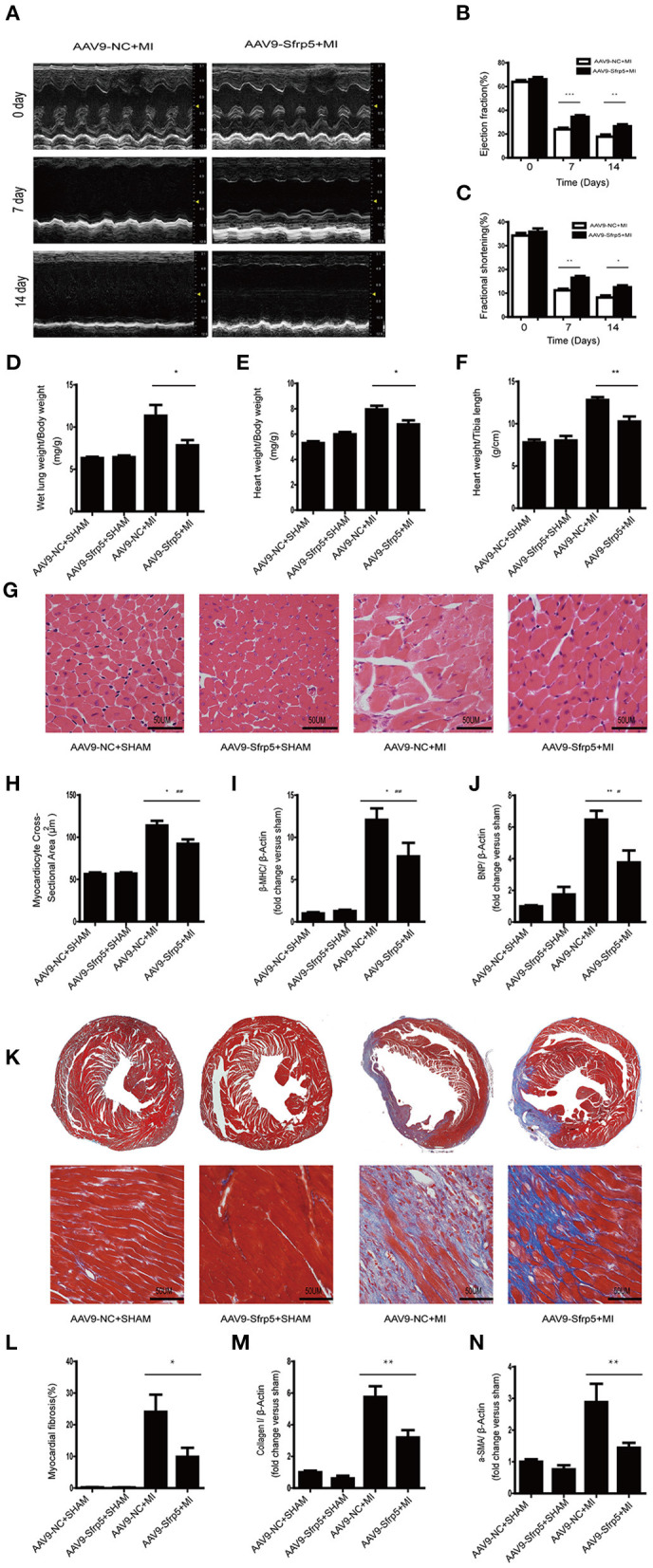
Sfrp5 improved cardiac function and alleviated cardiac remodeling at 2 weeks after MI. **(A)** Echocardiography of mice at 2 weeks post infarction (*n* = 9 per group). **(B)** LVEF analysis before and after MI. **(C)** LVFS analysis before and after MI. **(D)** Ratio of LW to BW (*n* = 6 per group). **(E)** Ratio of HW to BW (n = 6 per group). **(F)** Ratio of HW to TL (*n* = 6 per group). **(G)** Representative images of hearts stained with HE (*n* = 6 per group). Scale bar = 50 μm. **(H)** Myocardiocyte cross-sectional area of the heart at 2 weeks post infarction (*n* = 6 per group). **(I)** Cardiac β-MHC mRNA expression (*n* = 6 per group). β-Actin was used as a loading control. **(J)** Cardiac BNP mRNA expression (*n* = 6 per group). β-Actin was used as a loading control. **(K)** Representative images of hearts stained with Masson trichrome. Collagen deposits were stained blue (*n* = 6 per group). Scale bar = 50 μm. **(L)** Quantitative data of myocardial fibrosis in hearts after MI. **(M)** Cardiac collagen I mRNA expression (*n* = 6 per group). β-Actin was used as a loading control. **(N)** α-SMA mRNA expression (*n* = 6 per group). β-Actin was used as a loading control (values are presented as mean ± SD, *n* = 6 per group). *p < 0.05 AAV9-NC-MI mice at 14 days after MI vs. AAV9-Sfrp5-MI mice at 14 days after MI. **p < 0.01 AAV9-NC-MI mice at 7 or 14 days after MI vs. AAV9-Sfrp5-MI mice at 7 or 14 days after MI. ***p < 0.001 AAV9-NC-MI mice at 7 or 14 days after MI vs. AAV9-Sfrp5-MI mice at 7 or 14 days after MI. ^#^*p* < 0.05 AAV9-Sfrp5-MI mice at 14 days after MI vs. AAV9-Sfrp5-SHAM mice at 14 days after sham operation. ^##^*p* < 0.01 AAV9-Sfrp5-MI mice at 14 days after MI vs. AAV9-Sfrp5-SHAM mice at 14 days after sham operation.

We then examined the benefit of the decreased ischemic injury in the AAV9-Sfrp5 mice. Post-infarct ventricular remodeling in AAV9-NC and AAV9-Sfrp5 mice was assessed with serial echocardiography. Before MI, AAV9-NC and AAV9-Sfrp5 hearts had comparable chamber dimensions and ventricular function, but as early as 1 week post MI, AAV9-NC animals had a significantly greater decrease in LV ejection fraction (LVEF) and LV fractional shortening (LVFS) in comparison with AAV9-Sfrp5 animals, reflecting marked LV dysfunction in the AAV9-NC (Figures 3A–C). AAV9-Sfrp5 showed improved heart function compared with AAV9-NC up to the termination of the study (14 days). There were no significant differences at baseline between AAV9-NC and AAV9-Sfrp5. At 14 days post MI, LW/BW, HW/BW, and HW/TL were significantly reduced in AAV9-Sfrp5 mice, suggesting decreased post-MI HF and hypertrophy in AAV9-Sfrp5 hearts (Figures 3D–F). To examine hypertrophy at the cellular level, we measured the cross-sectional area of HE-stained LV sections. The cross-sectional area was less increased in AAV9-Sfrp5 cardiomyocytes, confirming decreased hypertrophic remodeling in the AAV9-Sfrp5 (Figures 3G,H). Next, we examined the re-expression of fetal genes as a marker of pathological hypertrophy and found less higher levels of β-myosin heavy chain at 14 days post-MI in AAV9-Sfrp5 mice, further confirming decreased pathological remodeling in the AAV9-Sfrp5 mice (Figure 3I). In addition, B-type natriuretic peptide, a biomarker of HF, was also less increased in the AAV9-Sfrp5 hearts (Figure 3J), consistent with the LW/BW data above.

The extent of myocardial fibrosis was determined by using Masson trichrome staining of histological sections at 2 weeks post MI. We observed a lesser increase in fibrosis in the border region in the AAV9-Sfrp5 hearts (Figures 3K,L). Next, we isolated RNA from the border myocardium 2 weeks post MI for quantification by real-time PCR of collagen-1 and α-SMA gene expression. Expressions of collagen-1 and α-SMA were less increased in AAV9-Sfrp5 hearts (Figures 3M,N). The lesser increase in collagen-1 and α-SMA gene expression is consistent with the lesser fibrosis seen in the AAV9-Sfrp5 hearts.

### AAV9-Sfrp5 Decreased Apoptosis, Suppressed Inflammation, and Improved Mitochondrial Dysfunction

**Figure 4:**
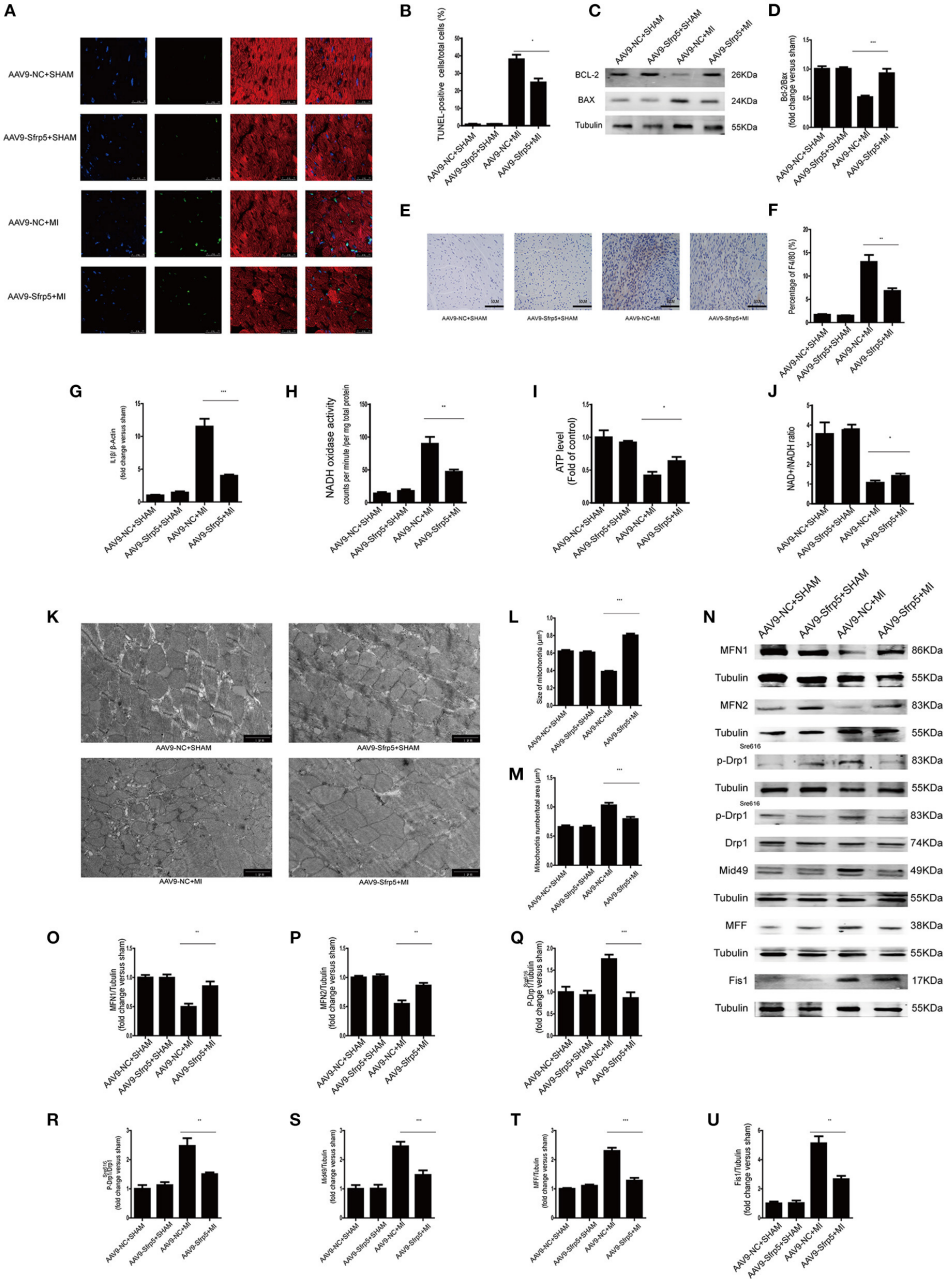
Sfrp5 decreased apoptosis and improved mitochondrial dysfunction. **(A)** Cardiomyocyte apoptosis determined by TUNEL staining. Scale bars = 25 μm. **(B)** Statistical analysis of TUNEL-positive cells (values are presented as mean ± SD, *n* = 6 per group). **(C)** Protein expression of Bax and Bcl-2 in mouse hearts at 14 days after MI. **(D)** Quantitative analysis of the relative Bcl-2/Bax ratio (*n* = 5 per group). **(E)** Macrophage infiltration determined by immunohistochemistry. Scale bars = 50 μm. **(F)** Statistical analysis of F4/80-positive cells (values are presented as mean ± SD, *n* = 6 per group). **(G)** IL1β mRNA expression (*n* = 6 per group). β-Actin was used as a loading control (values are presented as mean ± SD, *n* = 6 per group). (**H**) Quantitative analysis of the NADH oxidase activity level (values are presented as mean ± SD, *n* = 6 per group). **(I)** Quantitative analysis of the ATP level (values are presented as mean ± SD, *n* = 6 per group). **(J)** Quantitative analysis of the NAD^+^/NADH ratio (values are presented as mean ± SD, *n* = 5–6 per group). **(K)** Mitochondrial morphology was detected by TEM. Scale bars = 1 μm. **(L)** Quantitative analysis of the size of the mitochondria (values are presented as mean ± SD, *n* = 6 per group). **(M)** Quantitative analysis of the mitochondrial number/total area (values are presented as mean ± SD, *n* = 6 per group). **(N)** Protein expression of mitochondrial fusion proteins (MFN1 and MFN2) and mitochondrial fission proteins (p-Drp1^Ser616^, p-Drp1^Ser616^/Drp, Mid49, MFF, and Fis1) in mouse hearts at 14 days after MI (*n* = 5 per group). **(O)** Quantitative analysis of the relative MFN1 protein expression. Tubulin was used as a loading control. **(P)** Quantitative analysis of the relative MFN2 protein expression. Tubulin was used as a loading control. **(Q)** Quantitative analysis of the relative p-Drp1^Ser616^. Tubulin was used as a loading control. **(R)** Quantitative analysis of the relative p-Drp1^Ser616^/Drp. **(S)** Quantitative analysis of the relative Mid49 protein expression. Tubulin was used as a loading control. **(T)** Quantitative analysis of the relative MFF protein expression. Tubulin was used as a loading control. **(U)** Quantitative analysis of the relative Fis1 protein expression. Tubulin was used as a loading control. *p < 0.05 AAV9-NC-MI mice at 14 days after MI vs. AAV9-Sfrp5-MI mice at 14 days after MI. **p < 0.01 AAV9-NC-MI mice at 14 days after MI vs. AAV9-Sfrp5-MI mice at 14 days after MI. ***p < 0.001 AAV9-NC-MI mice at 14 days after MI vs. AAV9-Sfrp5-MI mice at 14 days after MI.

We next asked whether decreased cell death in the AAV9-Sfrp5 mice could be contributing to the improved cardiac function after MI. To determine the extent of apoptosis, TUNEL-positive nuclei were counted in the border region. Only cells that were both TUNEL positive and α-sarcomeric actin positive (cardiomyocyte marker), with TUNEL colocalizing with 4,6-diamidino-2-phenylindole, were counted as apoptotic cardiomyocytes. Cardiomyocyte apoptosis was comparable in the AAV9-Sfrp5 and AAV9-NC mice subjected to sham MI surgery. The number of TUNEL-positive myocytes was significantly lower in AAV9-Sfrp5 mice than in AAV9-NC mice after MI (Figures 4A,B). Thus, AAV9-Sfrp5 significantly decreased apoptosis in the border myocardium of the heart post-MI, including upregulation of the Bcl-2/Bax ratio (Figures 4C,D). To investigate whether Sfrp5 attenuates hypoxia-induced cardiomyocyte apoptosis by inhibiting inflammatory reaction, we examined macrophage infiltration with F4/80 antibody by using immunohistochemistry and quantified the expression of IL1β gene in real-time PCR. The infiltration degree of macrophages was significantly decreased in heart tissues at 14 days after MI in AAV9-Sfrp5 mice, and the expression of IL1β was less increased in AAV9-Sfrp5 hearts (Figures 4E–G). To explore whether Sfrp5 inhibited inflammatory reaction by improving mitochondrial function, we examined the mitochondrial function structure at 14 days after MI in the border zone. There was a marked decreased in NADH oxidase activity and increased ATP content as well as NAD^+^/NADH ratio in the myocardial mitochondria of AAV9-Sfrp5 mice (Figures 4H–J). Furthermore, the individual mitochondrial cross-sectional area was significantly greater, and the number of mitochondria per area was significantly lesser at 14 days after MI in AAV9-Sfrp5 mice (Figures 4K–M). Compared with the AAV9-NC mice, mitochondrial fusion proteins (MFN1/MFN2) were unregulated in the AAV9-Sfrp5 mice after MI, and mitochondrial fission proteins (p-Drp1^Ser616^/Mid49/MFF/Fis-1) were downregulated (Figures 4N–U).

**Figure 5:**
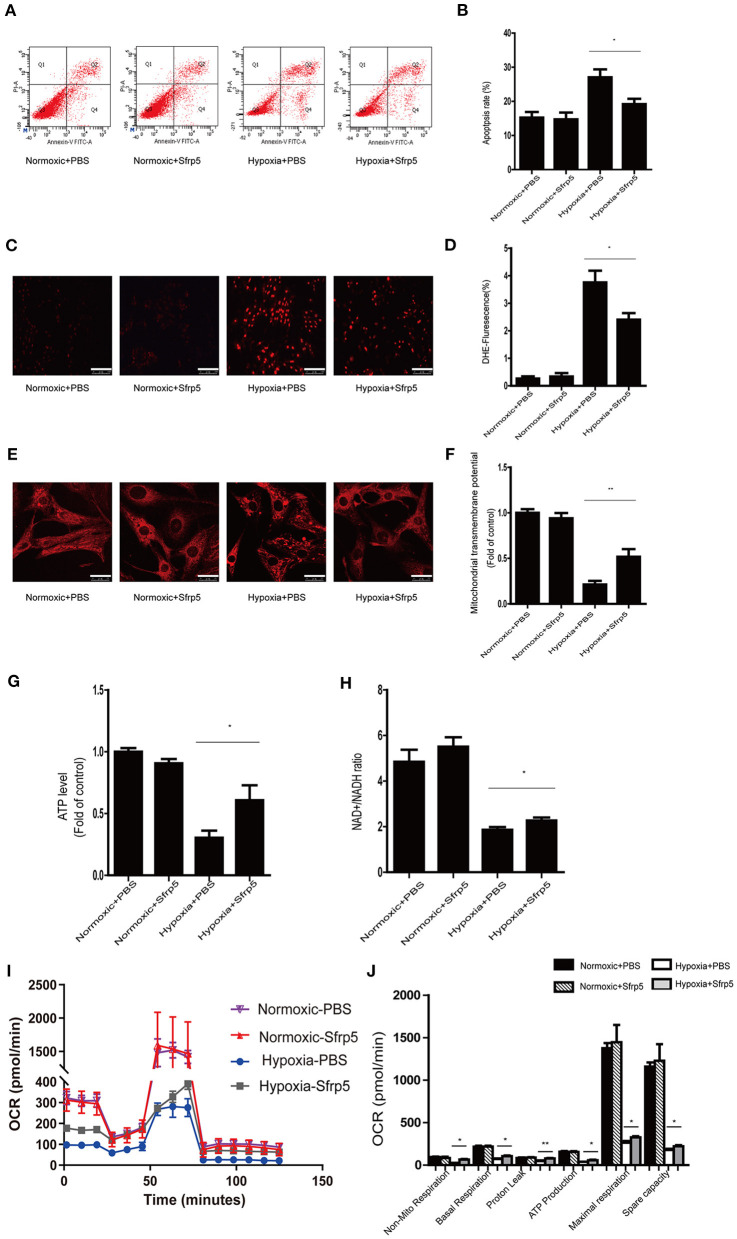
Sfrp5 treatment attenuated hypoxia-induced myocardial oxidative stress and mitochondrial dysfunction *in vitro*. **(A)** The rate of apoptosis was detected by flow cytometry. **(B)** Statistical analysis of apoptosis calculated from the flow cytometry in **A**. **(C)** Representative images of DHE staining of PBS-treated and Sfrp5-treated neonatal rat cardiac myocyte cells under normoxia or hypoxia conditions. Scale bars = 100 μm. **(D)** Statistical analysis of DHE-positive neonatal rat cardiac myocyte cells. **(E)** Mitochondrial morphology was detected by Mito-Tracker staining. Scale bars = 25 μm. **(F)** Quantitative analysis of mitochondrial transmembrane potential. **(G)** Quantitative analysis of the ATP level. **(H)** Quantitative analysis of the NAD^+^/NADH ratio. **(I)** Representative images of OCRs in PBS-treated and Sfrp5-treated neonatal rat cardiac myocyte cells under normoxia or hypoxia conditions as analyzed by the Seahorse XF. **(J)** Quantitative analysis of mitochondrial respiration calculated from the OCR trace in **G**. *p < 0.05 Hypoxia+PBS group vs. Hypoxia+Sfrp5. **p < 0.01 Hypoxia+PBS group vs. Hypoxia+Sfrp5.

To further explore whether Sfrp5 plays a protective role in cell apoptosis and mitochondrial dysfunction after MI in an *ex vivo* setting, the apoptosis rate, ROS production, and mitochondrial membrane potential were measured in cardiomyocytes treated with Sfrp5. Sfrp5 significantly decreased the rate of cell apoptosis following hypoxia treatment (Figures 5A,B). In addition, Sfrp5 reduced ROS production and increased mitochondrial transmembrane potential in hypoxia cardiomyocytes (Figures 5C–F). ATP content and NAD^+^/NADH ratio evaluated by fluorometric or colorimetric methods showed that treatment with Sfrp5 significantly improved hypoxia-induced mitochondrial dysfunction compared to those in the PBS-treated group (Figures 5G,H). Moreover, the mitochondrial respiratory chain function tested by OCR measurements suggested that Sfrp5-treated cardiomyocyte has significantly higher levels of nonmitochondrial respiration, basal respiration, proton leak, ATP production, maximal respiration, and spare capacity than that treated with PBS after hypoxia (Figures 5I,J). Sfrp5 decreased hypoxia-induced cardiomyocyte apoptosis, at least in part, via disrupting mitochondrial dysfunction.

### Sfrp5 Preserves Mitochondrial Dysfunction Partly via AMPK Pathway

**Figure 6:**
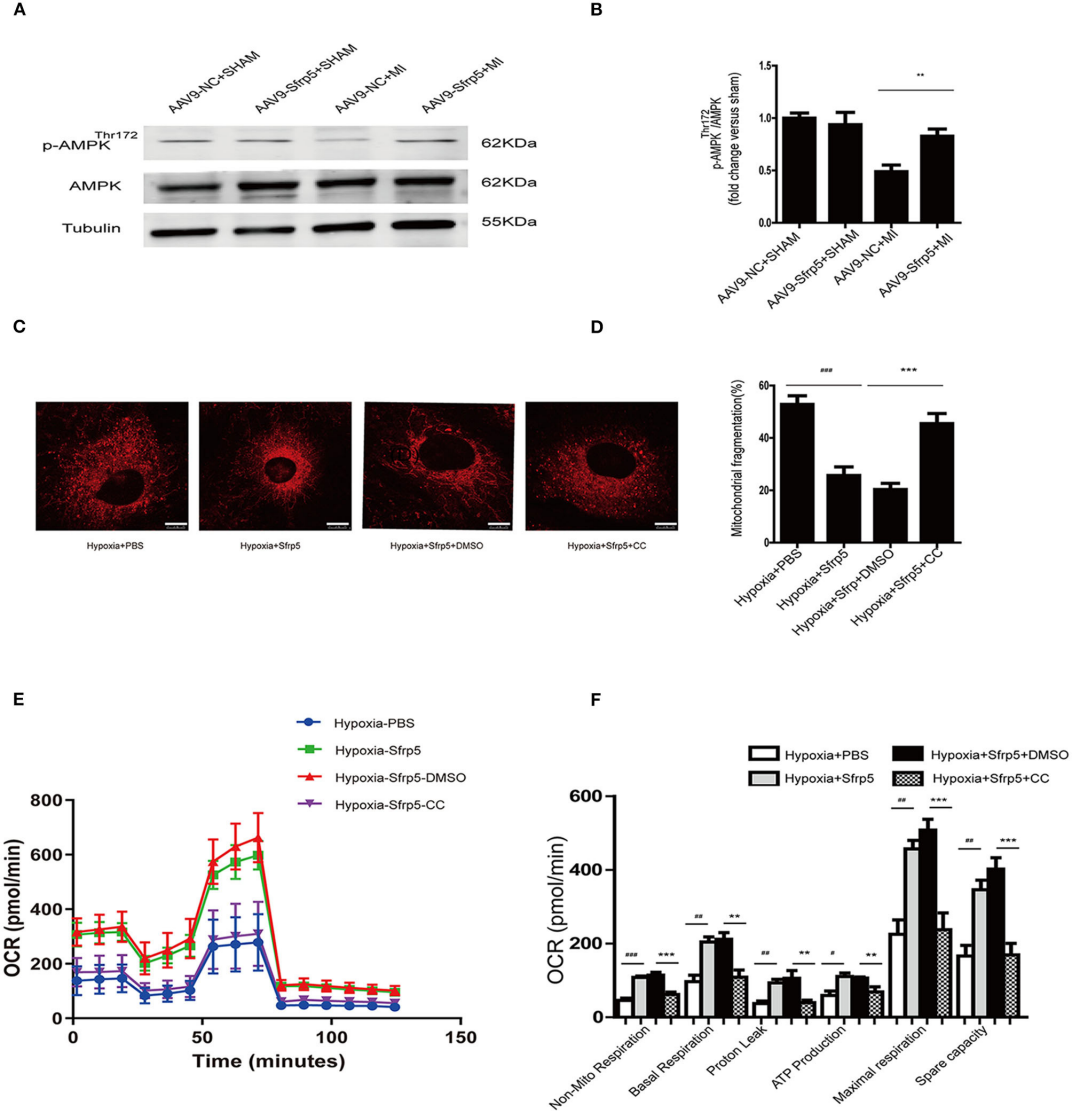
Sfrp5 preserves mitochondrial function partly through the AMPK pathway. **(A)** Protein expression of AMPK in mouse hearts at 14 days after MI (*n* = 4 per group). **(B)** Quantitative analysis of the relative p-AMPK^Thr172^/AMPK. **(C)** Mitochondrial morphology was detected by Mito-Tracker staining. Scale bars = 10 μm. **(D)** Quantitative analysis of mitochondrial fragmentation. **(E)** Representative images of OCR in Sfrp5-treated, PBS-treated, Sfrp5+Compound C-treated and Sfrp5+DMSO-treated neonatal rat cardiac myocyte cells under hypoxia conditions as analyzed by the Seahorse XF. **(F)** Quantitative analysis of mitochondrial respiration calculated from the OCR trace in **E**. ^**^p < 0.01 AAV9-NC+MI mice at 14 days after MI vs. AAV9-Sfrp5+MI mice at 14 days after MI. ^#^*p* < 0.05 Hypoxia+PBS group vs. Hypoxia+Sfrp5. ^##^*p* < 0.01 Hypoxia+PBS group vs. Hypoxia+Sfrp5. ^###^*p* < 0.001 Hypoxia+PBS group vs. Hypoxia+Sfrp5. **p < 0.01 Hypoxia+Sfrp5+DMSO group vs. Hypoxia+Sfrp5+CC. ***p < 0.001 Hypoxia+Sfrp5+DMSO group vs. Hypoxia+Sfrp5+CC.

To evaluate this further, we investigated the signaling pathway implicated in regulating mitochondrial biogenesis and found AMPK (Figures 6A,B) with phospho-AMPK being increased almost 25% of that of the AAV9-NC-treated level. To further clarify the roles of AMPK, we performed the experiments using neonatal rat cardiac myocytes. We conducted the Mito-Tracker staining experiment to explore the role of Sfrp5 on mitochondrial morphology and its modulation by AMPK in mitochondrial dynamics (fusion and fission). The cells were divided into four groups as follows: hypoxia+PBS, hypoxia+Sfrp5, hypoxia+Sfrp5+DMSO, and hypoxia+Sfrp5+Compound C. The results showed that Sfrp5 decreased mitochondrial fragmentation, while inhibition of AMPK with compound C abolished the benefits of Sfrp5-induced mitochondrial protection under hypoxia (Figures 6C,D). The results of OCR showed that the benefit of Sfrp5 on mitochondrial respiratory function in hypoxia cardiomyocytes including nonmitochondrial respiration, basal respiration, ATP production, proton leak, maximal respiration, and spare capacity were dismissed after treatment with compound C (1 mM) (Figures 6E,F). This would be expected to ameliorate ischemia-induced cell death by improving mitochondrial dysfunction via the AMPK signaling pathway.

**Figure 7:**
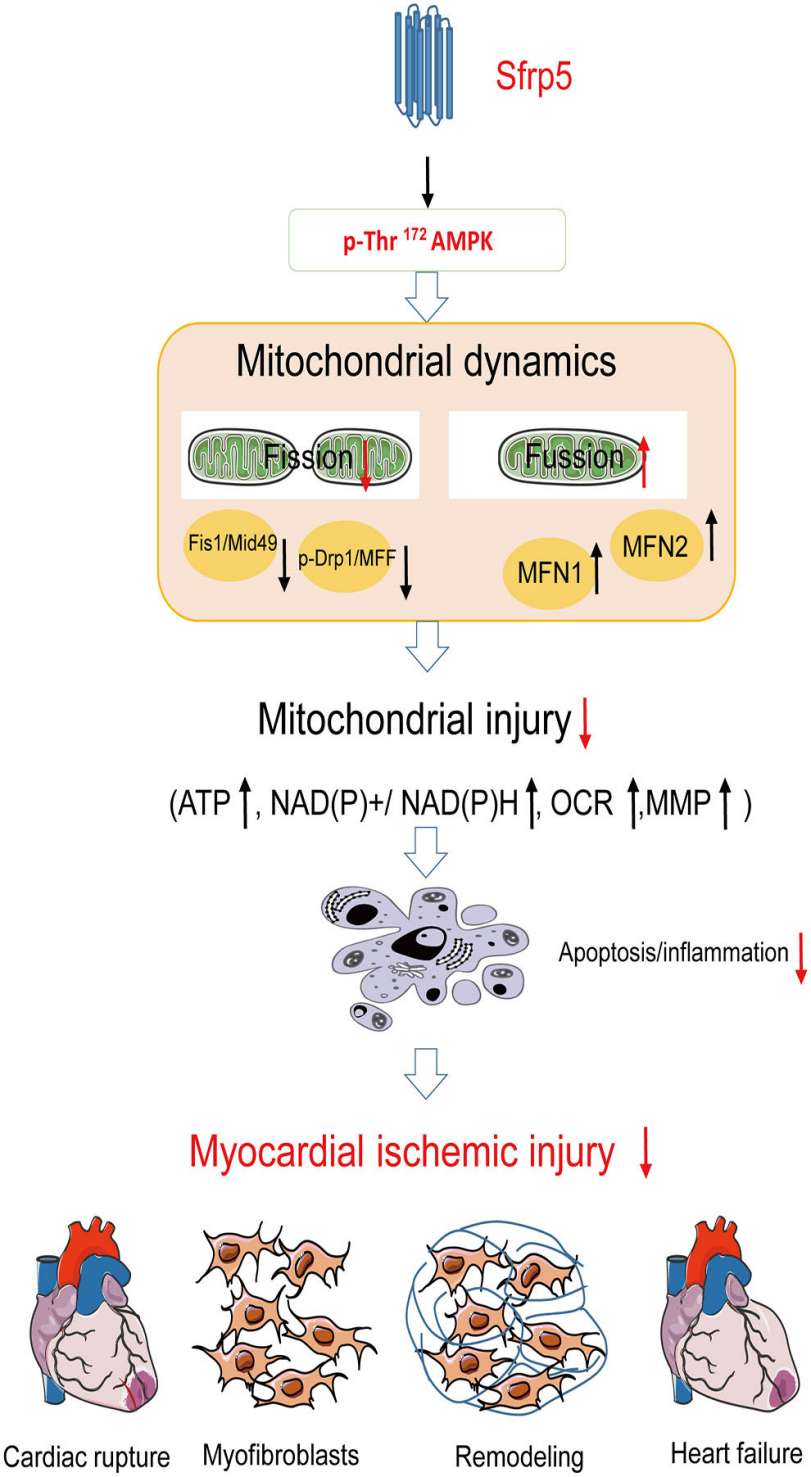
Summary of Sfrp5 effects and the proposed mechanism of Sfrp5 effect on MI and mitochondrial dysfunction. Sfrp5 protected against ischemic injury and attenuated cardiac remodeling through mitochondrial function improvement partly via activating the AMPK signaling.

Taken together, in the present study, we found that Sfrp5 may protect against ischemic injury and attenuated cardiac remodeling through mitochondrial function improvement partly via AMPK signaling (Figure 7).

## Discussion

In the present study, we found that the post-MI cardiac remodeling was closely associated with Sfrp5 downregulation. Overexpression of myocardial Sfrp5 attenuated cardiac dysfunction, decreased myocardial oxidative stress, suppressed inflammation, reduced cardiac fibrosis, and inhibited cardiomyocyte death. At the molecular levels, Sfrp5 overexpression improved mitochondrial dysfunction by decreasing the rate of apoptosis, reducing ROS production, enhancing mitochondrial respiration, and maintaining mitochondrial potential reduction. Further analysis identified that Sfrp5 improved mitochondrial dysfunction through mediating mitochondrial fission/fusion partly via the AMPK pathway. Inhibition of AMPK activity abrogated the beneficial effect of Sfrp5 on mitochondrial function. Altogether, our results identify that Sfrp5 plays a protective role in post-MI cardiac remodeling via disruption of mitochondrial dysfunction and partly via normalization of the AMPK pathway.

The Wnt signaling pathway is an elaborate and complex signal transduction pathway best recognized for regulating developmental processes including cell proliferation, differentiation, and tissue patterning (20). In the adult heart, the Wnt pathway is thought to be quiescent but is reactivated during cardiac injury. Excessive Wnt pathway activity has an adverse effect on cardiovascular pathology and aborts regeneration of damaged cardiac tissue (21, 22). Inhibition in abnormal activation of the Wnt signaling pathway reduces infarct expansion and prevents the development of HF after MI (23). Soluble frizzled-related proteins (Sfrps) as an antagonist for the Wnt pathway were shown to attenuate LV remodeling following myocardial injury (24). Sfrps contain five members, namely, Sfrp1, Sfrp2, Sfrp3, Sfrp4, and Sfrp5. Previous studies have shown the protective effects of Sfrps member in the progression of cardiovascular disease (25–27). Among them, Sfrp1 was shown to attenuate LV remodeling and prevent cardiac rupture following myocardial injury (27). Sfrp2 was demonstrated to improve cardiac function and resist myocardial fibrosis progression after MI (25). Sfrp3 and Sfrp4 were both proven to be closely associated with HF (28). But, to our knowledge, only one report has addressed the effect of Sfrp5 on myocardial ischemia injury. The authors of that study used Sfrp5-knockdown mice and concluded that the loss of Sfrp5 induced larger infarct sizes, caused extravagant inflammation reaction, increased cardiomyocyte apoptosis, and decreased cardiac function following ischemia/reperfusion injury (14). However, the exact role of Sfrp5 during the chronic phase of MI is still unclear. Recently, two relative studies have suggested that Sfrp5 reduced myofibroblast growth and ameliorated cardiomyocyte hypertrophy (29, 30). Consistent with those studies, we found that overexpression of Sfrp5 decreased myocardial fibrosis and inhibited myocardial hypertrophy in mice after MI. We further demonstrated the protective effects of Sfrp5 overexpression on the reduction of the MI area and LV remodeling as demonstrated by the decreased risk of cardiac rupture and dysfunction in mice with MI injury.

The reduced apoptosis and improved mitochondrial dysfunction seen in AAV9-Sfrp5 mice probably played a major role in attenuating the cardiac rupture rate and limiting the cardiac remodeling post MI (31). The role of the Wnt/fz pathway in programmed cell death is a matter of debate. Sfrps were first related to apoptosis, thus making cells more prone to apoptosis under apoptotic conditions (32). However, in recent reports, it was shown that Sfrp5 decreased the apoptotic rate in myocardium during ischemia–reperfusion injury *in vivo* through limitation of inflammatory cell infiltration (14). The protective effect of Sfrp5 against cell death post MI in mice could be different from that of ischemia–reperfusion injury. In AAV9-Sfrp5 mice at 14 days after MI, the individual mitochondrial cross-sectional area was significantly greater, and the number of mitochondria per area was significantly lesser than those in AAV9-NC mice, as observed by electron microscopy. It is now admitted that the mitochondrion was a critical mediator of cell metabolism and survival, and its abnormalities play a key role in the post-MI process (33). It has recently been shown that Sfrp5 participates in mitochondrial metabolic dysfunction (8). Sfrp5 was shown to suppress oxidative metabolism through decreasing adipocyte mitochondrial function in adipocyte. However, during myocardial ischemia, decreased mitochondrial function contributes to cardiac dysfunction and cardiomyocyte apoptosis via loss of metabolic capacity as well as ATP production and release of toxic products (3). Preservation of mitochondrial function is shown to be a novel therapeutic strategy in the setting of MI. Apoptosis-mediated cardiomyocyte cell is regulated by pro-apoptotic (Bax) and anti-apoptotic proteins (Bcl-2) (34). Bcl-2/Bax ratio imbalance in return initiates apoptosis through destruction of mitochondrial membrane integrity. Loss of mitochondrial membrane integrity leads to the production of ROS and deteriorates the process of post-MI cardiac remodeling and therefore increases the risk of cardiac rupture (35). We found that the Bcl-2/Bax ratio was significantly higher in AVV9-Sfrp5 mice than in AAV9-NC. Moreover, overexpression of Sfrp5 increased ATP generation as well as NAD^+^/NADH ratio and decreased NADH oxidase activity. In parallel, Sfrp5 treatment improved the mitochondrial respiratory function and mitochondrial membrane potential during hypoxia in NRCMs. These changes were mainly related to reduction of cardiomyocyte cell death and improvement of mitochondrial dysfunction as suggested previously (3). We next analyze the effect of Sfrp5 on mitochondrial dynamics proteins including fusion-related and fission-related proteins.

Mitochondrial fission and fusion are crucial processes in regulating mitophagy and the dynamics of the mitochondrial network (36). Excessive mitochondrial fission and decreased mitochondrial fusion lead to mitochondrial dysfunction, decreasing the oxidative capacity of cardiomyocyte (37). We found that Sfrp5 overexpression decreased the number of mitochondria per area, raised the individual mitochondrial cross-sectional area, increased the expression of mitochondrial fusion proteins (MFN1 and MFN2), and decreased the expression of mitochondrial fission proteins (p-Drp1^Ser616^/Mid49/MFF/Fis-1). It is reasonable to consider that Sfrp5 balances the fission and fusion of mitochondria to benefit mitochondrial dynamics. We further investigate the exact mechanism of Sfrp5 in improving mitochondrial dysfunction.

AMPK has been suggested as an important regulator of mitochondrial metabolism (38). Activated AMPK represses Drp1 phosphorylation, attenuates mitochondrial fission, and therefore prevents cardiac injury from occurring due to myocardial ischemia (37). LKB1 is the major upstream kinase of AMPK mediating AMPK phosphorylation in the cardiac tissues (39). Cardiomyocyte-specific knockout of LKB1 would cause cardiac hypertrophy through inhibition of AMPK signaling in mice (40). Sirtuin 1 (SIRT1) could activate the downstream AMPK by diminishing the lysine acetylation of LKB1. A previous study showed that SIRT1 regulated the expression of Wnt signaling antagonists Sfrp1 and Sfrp2 (41, 42). We found that Sfrp5 overexpression activated phosphorylation AMPK in MI-induced myocardial injury. Inhibition of AMPK by compound C mitigated the protective effect of Sfrp5. In addition, the expression of SIRT1 was significantly decreased over time in heart tissues of MI as detected by western blot, which is consistent with the change of Sfrp5 expression (Supplementary Figures 1G,H). Accordingly, our results, suggested that Sfrp5 might protect against cardiac remodeling through restoring mitochondrial function partly via the SIRT1-LKB1-AMPK signaling pathway. However, the direct target for Sfrp5 deserves further investigation.

## Conclusion

This study mainly revealed the protective effect of Sfrp5 after MI, and the potential mechanisms which improve mitochondrial function by partly activating the AMPK signaling.

## Data Availability Statement

The original contributions presented in the study are included in the article/Supplementary Materials, further inquiries can be directed to the corresponding author/s.

## Ethics Statement

The animal study was reviewed and approved by The Animal Subjects Committee of Capital Medical University.

## Author Contributions

WG and SN: conceptualization, writing—review & editing, and supervision. XH and YM: methodology and project administration. WZ: software. XW: validation. XH: formal analysis. YM: investigation. YY: data curation. XH: writing—original draft preparation. XH and YM: project administration. WG, XW, and SN: funding acquisition. All authors contributed to the article and approved the submitted version.

## Funding

This study was supported by grants from the Beijing Natural Science Foundation (7191002), National Natural Science Foundation of China (81970292 and 81870322), Beijing Hospitals Authority Youth Program (QML20190603), Capital's Funds for Health Improvement and Research (2018-1-2061), CS Optimizing Antithrombotic Research Fund (BJUHFCSOARF201901-08), Beijing Municipal Administration of Hospitals Clinical Medicine Development of Special Funding Support (ZYLX201710), Beijing Municipal Administration of Hospitals' Ascent Plan (DFL20180601), and Beijing Nova Program (Z201100006820087).

## Conflict of Interest

The authors declare that the research was conducted in the absence of any commercial or financial relationships that could be construed as a potential conflict of interest.

## Publisher's Note

All claims expressed in this article are solely those of the authors and do not necessarily represent those of their affiliated organizations, or those of the publisher, the editors and the reviewers. Any product that may be evaluated in this article, or claim that may be made by its manufacturer, is not guaranteed or endorsed by the publisher.
