# Supplementary figures and images for: Secreted Frizzled-Related Protein 5 Protects Against Cardiac Rupture and Improves Cardiac Function Through Inhibiting Mitochondrial Dysfunction
Source: Front Cardiovasc Med. 2021 Sep 9;8:682409. doi: 10.3389/fcvm.2021.682409 (PMC8458704; doi:10.3389/fcvm.2021.682409)

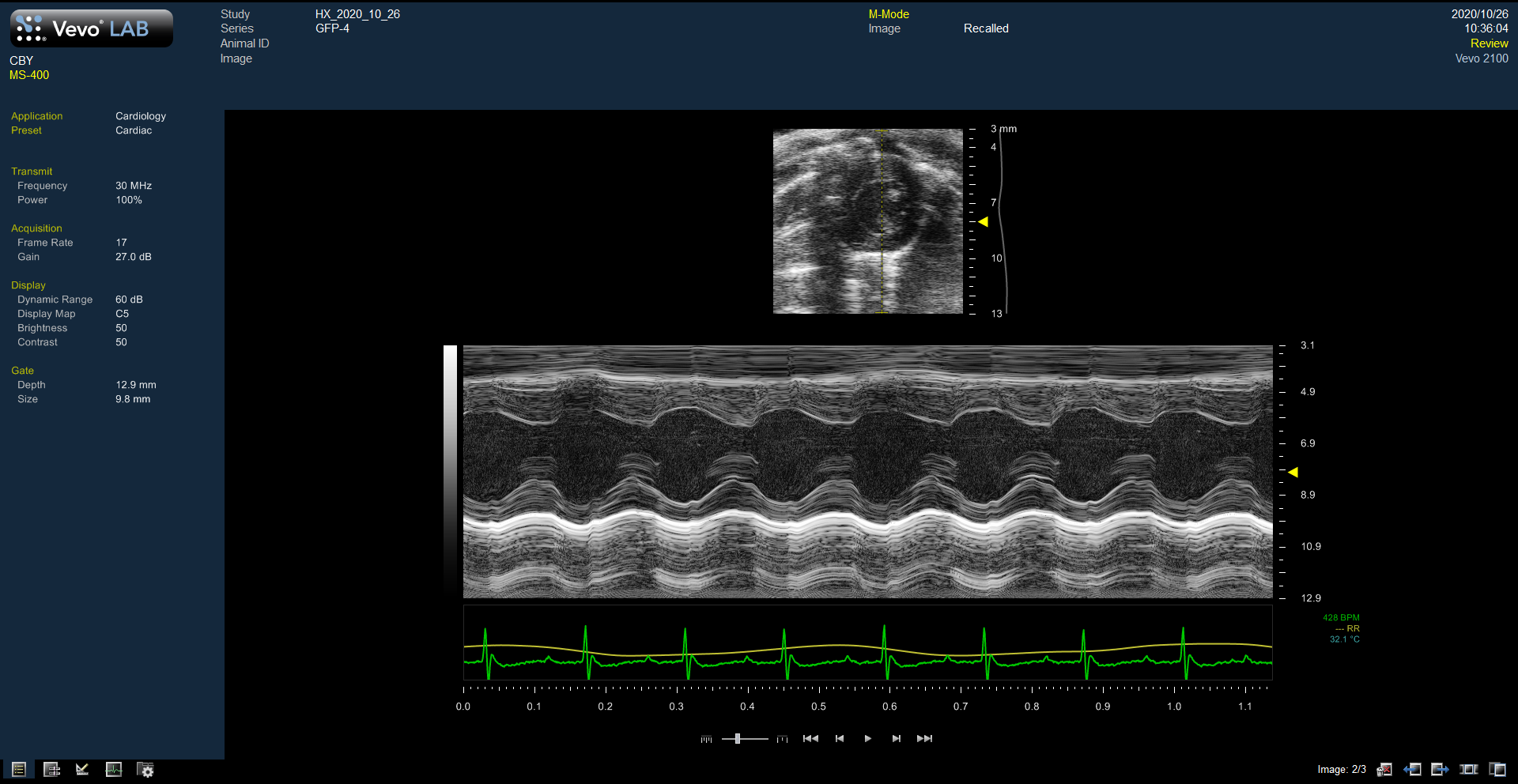

Supplement: Supplementary file 1 [file Data_Sheet_1.zip › sup/D-AAV9-NC.tif]

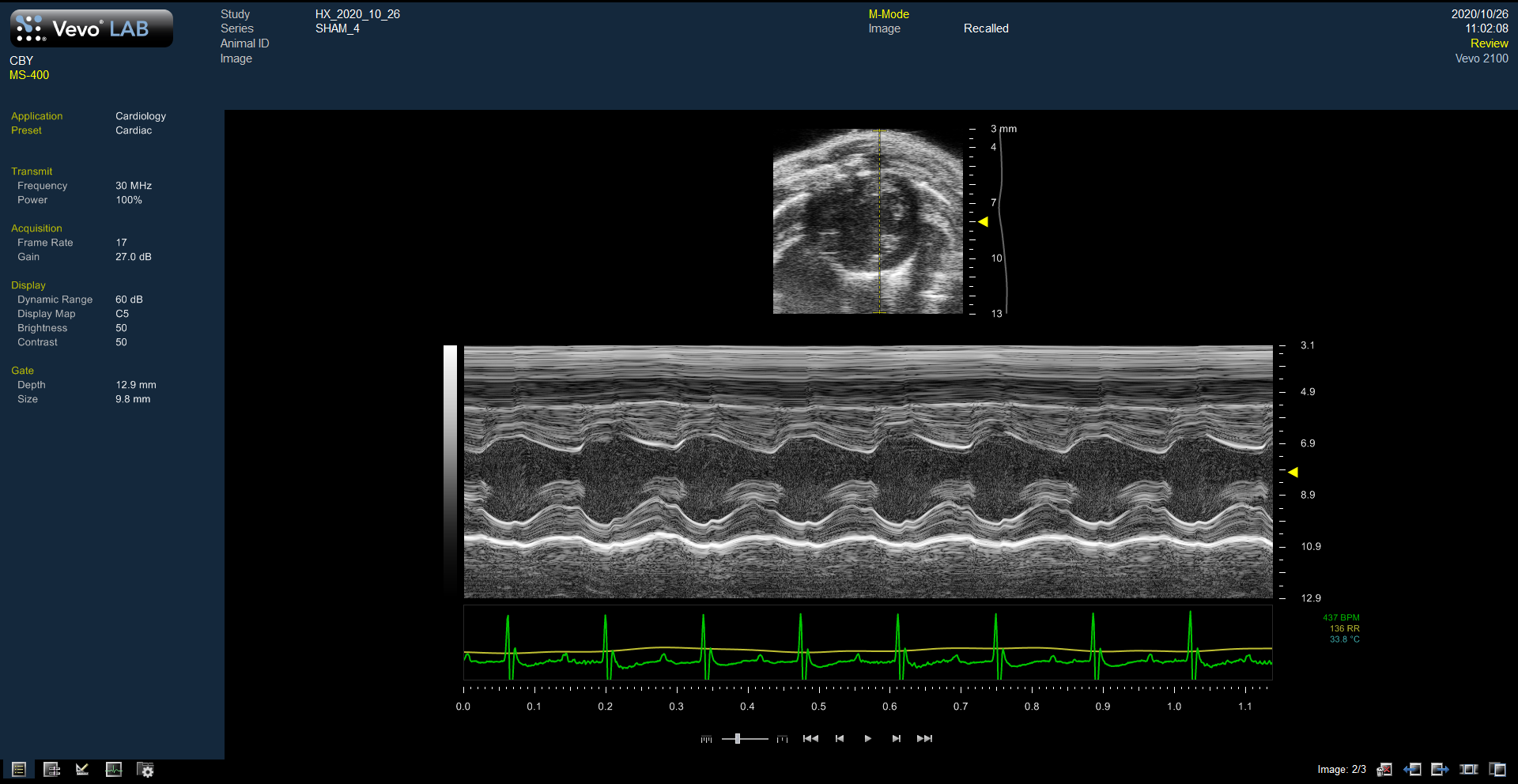

Supplement: Supplementary file 1 [file Data_Sheet_1.zip › sup/D-WT-SHAM.tif]

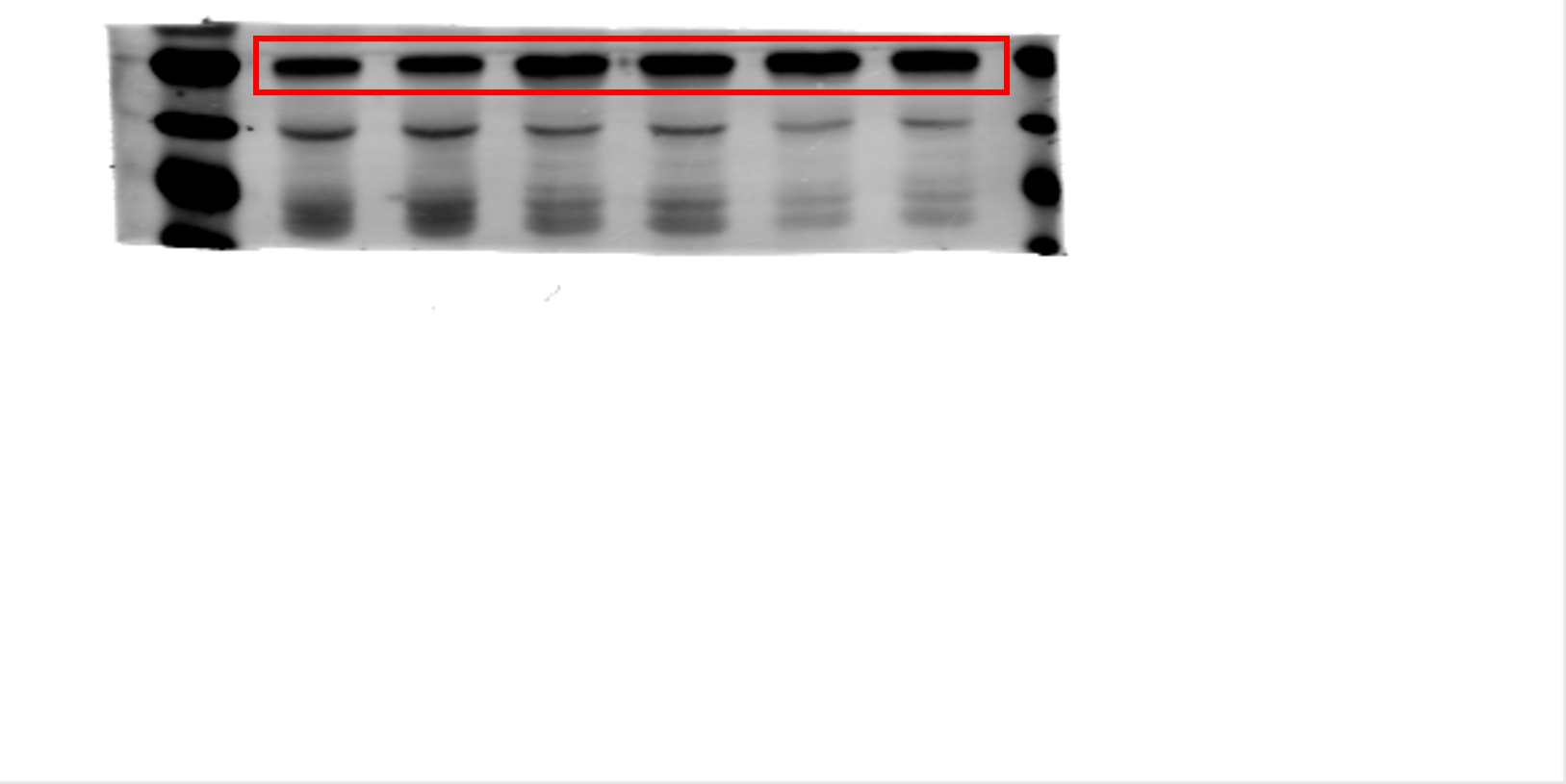

Supplement: Supplementary file 1 [file Data_Sheet_1.zip › sup/G-Tubulin .tif]
