# Supplementary material for: Secreted Frizzled-Related Protein 5 Protects Against Cardiac Rupture and Improves Cardiac Function Through Inhibiting Mitochondrial Dysfunction
Source: Front Cardiovasc Med. 2021 Sep 9;8:682409. doi: 10.3389/fcvm.2021.682409 (PMC8458704; doi:10.3389/fcvm.2021.682409)
Supplement: Supplementary file 2 [file Data_Sheet_2.docx]

Supplementary Material

**Supplemental Methods**

**1. The sequence of AAV9/Sfrp5**

ATGTGGGTGGCCTGGAGCGCACGGACGGCCGCACTGGCGTTGCTGCTCGGGGCGCTGCATGGGGCGCCAACACGCGGCCAGGAGTACGACTACTACGGTTGGCAGGCCGAGCCGCTGCACGGCCGCTCCTACTCCAAGCCACCGCAGTGCCTCGACATCCCCGCCGATCTGCCGCTCTGTCACACGGTGGGCTACAAGCGCATGCGGCTGCCCAACCTGCTGGAGCACGAGAGCCTGGCCGAGGTGAAGCAGCAGGCAAGCAGCTGGCTGCCACTGCTGGCCAAGCGCTGCCACTCAGACACCCAGGTCTTCCTCTGCTCGCTCTTCGCTCCCGTCTGCCTGGACCGACCCATCTACCCCTGCCGCTCGCTGTGCGAAGCCGTGCGCGCCGGCTGCGCTCCGCTCATGGAGGCCTACGGTTTCCCTTGGCCCGAGATGCTGCACTGCCACAAGTTCCCCCTGGACAACGACCTCTGCATCGCGGTGCAGTTCGGGCACCTGCCTGCCACCGCGCCTCCAGTGACCAAGATCTGTGCCCAGTGTGAGATGGAGCACAGCGCTGATGGCCTCATGGAACAGATGTGCTCCAGTGACTTTGTGGTCAAGATGCGCATTAAGGAGATCAAGATAGACAACGGGGACCGAAAGTTGATTGGAGCCCAGAAGAAGAAGAAGCTGCTCAAGGCAGGCCCCTTAAAGCGCAAGGACACCAAGAAGCTGGTCCTGCATATGAAGAACGGGGCAAGCTGCCCTTGCCCACAGTTAGACAACCTGACGGGCAGCTTCCTGGTCATGGGCCGCAAAGTAGAGGGACAGCTGCTGCTCACGGCCGTCTACCGCTGGGACAAGAAGAATAAGGAGATGAAGTTTGCAGTCAAATTCATGTTCTCCTATCCCTGTTCCCTCTACTACCCTTTTTTCTATGGGGCAGCTGAACCCCACTGA

**2. Elisa**

Blood was collected via venipuncture of the right ventricle and collected in heparin moistened tubes. Plasma was isolated, snap frozen and stored at −80 °C to analyze the concentrations of cardiac injury markers of CK-MB. The concentration of CK-MB was detected using a Creatine kinase MB isoenzyme Assay Kit (Jiancheng Bioengineering Institute, Nanjing, China, Cat. No: H197) according to the manufacturer’s instructions.

**3. Assessment of NADH oxidase activity activity**

We evaluated the NADH oxidase activity of mitochondria in heart tissues using commercially available kits (Solarbio, China, Cat. No: BC 0630), according to the manufacturer’s instructions. The level of superoxide anion after MI was detected by Dihydroethidium (Beyotime,S0063).

**4. Immunocytologic Staining**

NRCMs were stained for mitochondria by Mito-Tracker Red CMXRos (C1049, Beyotime). A stock solution at a concentration of 200 μmol/L was prepared in dimethyl sulfoxide and stored at -20°C. NRCMs were stained in complete DMEM/F12 cell-culture medium containing 0.5 mmol/L cell permeant Mito-Tracker Red CMXRos for 30 minutes at 37°C. After washing 3 times for 10 min each in PBS, they were fixed in 4% paraformaldehyde for 20 minutes at room temperature. The NRCMs were then exposed to fluorescence mounting medium with DAPI for 5 minutes to detect nuclei. After 3 washes in PBS, they were mounted on glass slides and observed by laser scanning confocal microscopy (Leica TCS SP2, Wetzlar Germany).

**5. TUNEL assay**

Paraffin section of LV was dewaxed in xylene, hydrated in gradient ethanol and the antigen of α-actinin was retrieved by citric acid buffer microwave antigen retrieval method. Specimens and slides were subsequently blocked with 10% goat serum albumin for 1 h at room temperature, incubated with primary antibodies-alpha actinin (ab9465, Abcam) overnight at 4°C, washed 3 times for 10 min each in PBS and incubated with secondary antibody (A32727, Thermo Fisher Scientific) for 1 hour at room temperature protecting from light. The terminal deoxynucleotidyl transferase UTP nick end-labeling (TUNEL) assay was conducted by using an In Situ Cell Death Detection Kit (Roche Diagnostics, 12156792910, Branford, CT, USA) according to the manufacturer’s instructions. After washing with PBS three times, the sections were mounted in fluorescence mounting medium with DAPI (Vector Laboratories, Inc. Burlingame, CA 94010) to determine the nuclei. All the paired sections were examined under a confocal laser scanning microscope.

**6. qPCR**

Total cellular RNA was extracted from ventricular tissue (20mg) using TRIzol reagent (Invitrogen, Carlsbad, CA), 5 mm stainless steel beads, and the multi-sample tissue grinder (OMNI Bead Ruptor, USA). Total cellular RNA was quantified using a NanoDrop ND8000 spectrophotometer and 1-2 μg total RNA were translated to cDNA using RevertAid First Strand cDNA Synthesis Kit(Thermo Fisher Scientific, Waltham, MA, K1622) according the kit’s protocol following the manuscript’s instruction. The resulting cDNA was performed to RT-PCR (CFX96Real-Time PCR Detection System) using SYBR® Premix Ex Taq™ (Tli RNaseH Plus) (TAKARA, DRR420A). Cq values and melt curves were analyzed with the CFX Manager 3.0.1224.1015. (Bio-Rad, USA). The qRT-PCR used primers were as follows:

|  |  |  |
| --- | --- | --- |
| Gene | Forward primer | Reverse primer |
| Sfrp5 | 5'- GAGATCAAGATAGACAACGGGGA -3' | 5'- TTGCGCTTTAAGGGGCCTG-3' |
| BNP | 5'-GAAGGTGCTGTCCCAGATGA-3' | 5'-CCAGCAGCTGCATCTTGAAT-3' |
| Collagen-1 | 5'-GAGTACTGGATCGACCCTAACCA-3' | 5'-GACGGCTGAGTAGGGAACACA-3' |
| α-SMA | 5'-GTCCCAGACATCAGGGAGTAA-3' | 5'-TCGGATACTTCAGCGTCAGGA-3' |
| IL-1β | 5'-CTTCCCCAGGGCATGTTAAG-3' | 5'-ACCCTGAGCGACCTGTCTTG-3' |
| β-MHC | 5'-ACTGTCAACACTAAGAGGGTCA-3' | 5'-TTGGATGATTTGATCTTCCAGGG-3' |
| β-actin | 5'- CCCAGCACAATGAAGATCAAGATCAT-3' | 5'-ATCTGCTGGAAGGTGTACAGCGA-3' |

β‐actin was used for normalization, and all relative mRNA expressions were quantified by ΔΔCt method. All results were carried out expressed as change in fold relative to control

**7. Flow cytometry**

The rate of apoptosis was conducted by using an Annexin V-FITC/PI apoptosis detection kit (BD Biosciences, 556547) according to the manufacturer’s instructions. NRCMs were seeded into 6-well plate. At 12 hours following treatment, the cells were digested with trypsin, washed 3 times in PBS and resuspended in 200μL binding buffer. Then, the cells were stained with 5 μL Annexin V-fluorescein isothiocyanate (FITC) and 5 μL propidium iodide (PI) for 15 min at room temperature in the dark. The analysis was performed by flow cytometry within 1 hour.

**8. Immuno-histochemistry**

Paraffin section of LV was dewaxed in xylene, hydrated in gradient ethanol and the antigen of F4/80 was retrieved by citric acid buffer microwave antigen retrieval method. Specimens and slides were subsequently blocked with 10% goat serum albumin for 1 h at room temperature, incubated with primary anti-mouse monoclonal antibodies against F4/80 (1:200 dilution, Abcam, ab16911) overnight at 4°C, washed 3 times for 10 min each in PBS and incubated with a biotinylated secondary antibody for 30 minutes at 37°C. All the sections were examined by NIS-ELEMENTS automatic program (Nikon) under ×400 magnifications.

Since there was no difference between the AAV9-NC and WT-SHAM hearts, we only select the AAV9-NC as controls (online-only Data Supplement Figure 1D-1F).


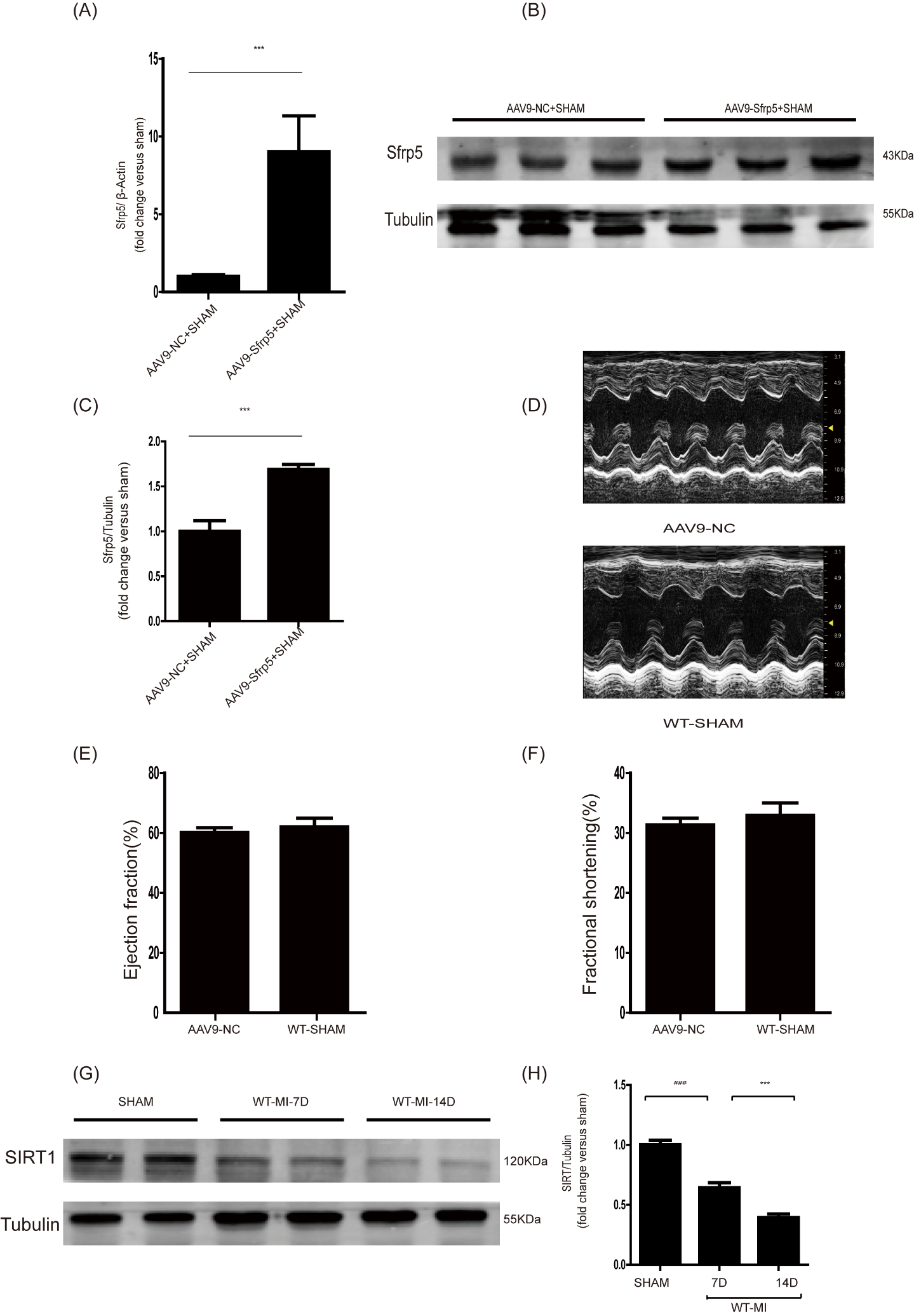


**Supplemental Figure 1**

(A) Protein expression of Sfrp5 in mouse hearts at 4 weeks after AVV9-NC/Sfrp5 injection by qPCR (n=6 per group). (B) Protein expression of Sfrp5 in mouse hearts at 4 weeks after AVV9-NC/Sfrp5 injection (n=6 per group). (C) Quantitative analysis of the relative Sfrp5 protein expression, Tubulin was used as a loading control. (D) Echocardiography of mice at 4 weeks after AAV9-Sfrp5 injection (E) Left ventricle ejection fraction analysis before myocardial infarction at 4 weeks afterAAV9-Sfrp5 (F) Left ventricle fractional shortening analysis before myocardial infarction at 4 weeks afterAAV9-Sfrp5. (values are presented as mean ± SEM, n=6 per group). (G) Protein expression of SIRT1 in mouse hearts at 7 and 14 days after myocardial infarction. (H) Quantitative analysis of the relative SIRT1 protein expression, Tubulin was used as a loading control (values are presented as mean ± SD, n=6 per group). ***P < 0.001 mice at 30 days after AVV9-NC injection vs. mice at 30 days after AVV9-Sfrp5 injection. ^###^P<0.001 mice at 7 days after myocardial infarction versus mice at 7 days after sham operation. ***P < 0.001 mice at 14 days after myocardial infarction versus mice at 7 days after myocardial infarction.
